# O-pH: Optical pH Monitor to Measure Dental Biofilm Acidity and Assist in Enamel Health Monitoring

**DOI:** 10.1109/TBME.2022.3153659

**Published:** 2022-08-19

**Authors:** Manuja Sharma, Lauren K. Lee, Matthew D. Carson, David S. Park, Se W. An, Micah G. Bovenkamp, Jess J. Cayetano, Ian A. Berude, Leonard Y. Nelson, Zheng Xu, Alireza Sadr, Shwetak N. Patel, Eric J. Seibel

**Affiliations:** Department of Electrical and Computer Engineering, University of Washington, USA.; Department of Microbiology, University of Washington, USA.; School of Dentistry, University of Washington, USA.; Department of Mechanical Engineering, University of Washington, USA.; School of Dentistry, University of Washington, USA.; School of Dentistry, University of Washington, USA.; School of Dentistry, University of Washington, USA.; School of Dentistry, University of Washington, USA.; School of Dentistry, University of Washington, USA.; Department of Mechanical Engineering, University of Washington, USA.; UW School of Dentistry, USA.; private dental practice in Seattle, WA, USA.; School of Dentistry, University of Washington, USA.; Department of Electrical and Computer Engineering, and Paul. G. Allen School of Computer Science, University of Washington, USA.; Department of Mechanical Engineering, Department of Bioengineering, Department of Electrical and Computer Engineering, School of Dentistry, University of Washington, Seattle, WA 98195 USA

**Keywords:** Fluorescein, fluorescence, ratiometric, caries, pH, plaque, oral biofilm, acidification, Stephan curve

## Abstract

**Objective:**

Bacteria in the dental biofilm produceacid after consumption of carbohydrates which if left unmonitored leads to caries formation. We present O-pH, a device that can measure dental biofilm acidity and provide quantitative feedback to assist in oral health monitoring.

**Method:**

O-pH utilizes a ratiometric pH sensing method by capturing fluorescence of Sodium Fluorescein, an FDA approved chemical dye. The device was calibrated to a lab pH meter using buffered fluorescein solution with a correlation coefficient of 0.97. The calibration was further verified *in vitro* on additional buffered solution, artificial, and extracted teeth. An *in vivo* study on 30 pediatric subjects was performed to measure pH before (rest pH) and after (drop pH) a sugar rinse, and the resultant difference in pH (diff pH) was calculated. The study enrolled subjects with low (Post-Cleaning) and heavy (Pre-Cleaning) biofilm load, having both unhealthy/healthy surfaces. Further, we modified point-based O-pH to an image-based device using a multimode-scanning fiber endoscope (mm-SFE) and tested *in vivo* on one subject.

**Results and Conclusion:**

We found significant difference between Post-Cleaning and Pre-Cleaning group using drop pH and diff pH. Additionally, in Pre-Cleaning group, the rest and drop pH is lower at the caries surfaces compared to healthy surfaces. Similar trend was not noticed in the Post-Cleaning group. mm-SFE pH scope recorded image-based pH heatmap of a subject with an average diff pH of 1.5.

**Significance:**

This work builds an optical pH prototype and presents a pioneering study for non-invasively measuring pH of dental biofilm clinically.

## I. INTRODUCTION

**Fig. 1.**
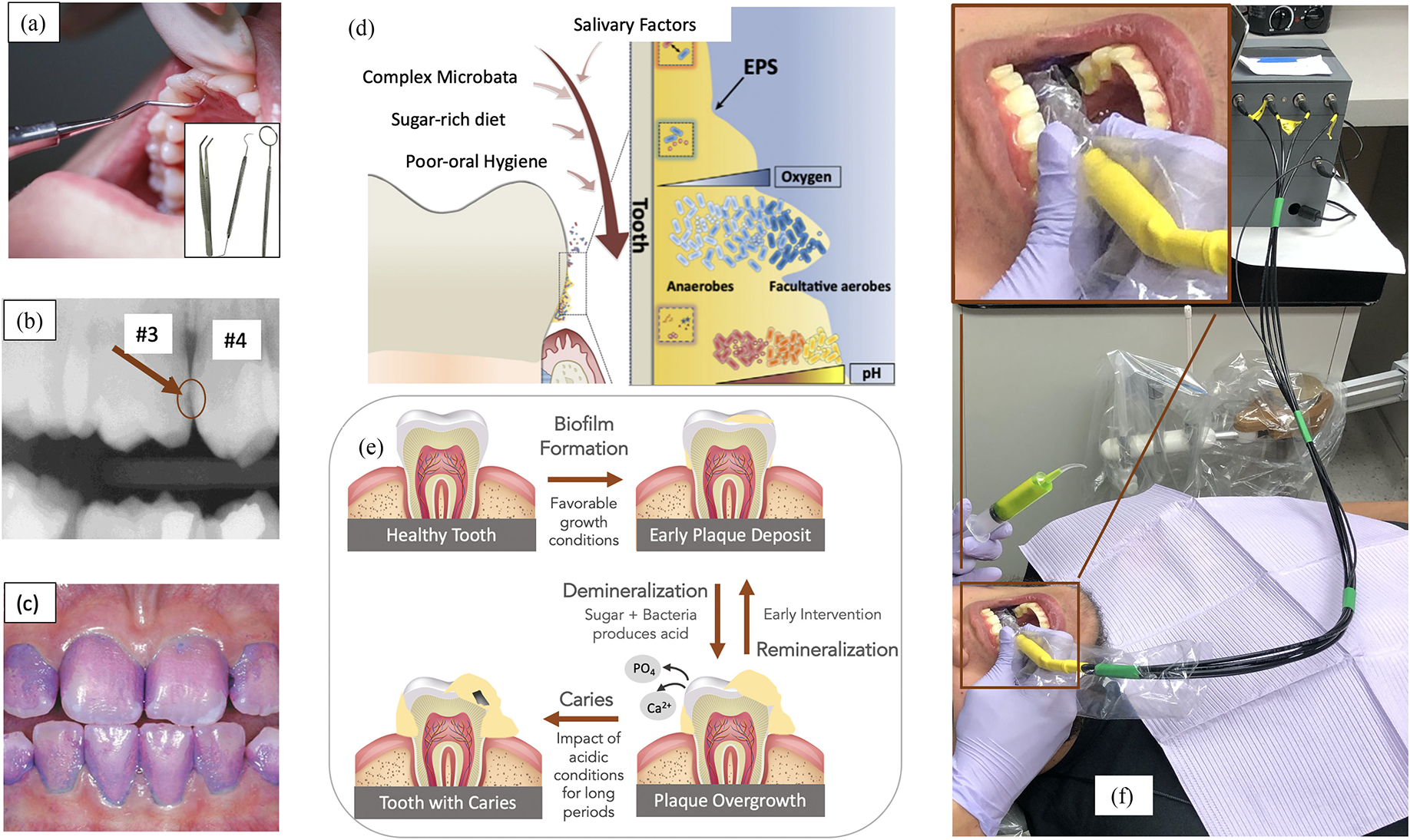
(a) Visual assessment using dental tools - gold standard for early occlusal caries. Inset figure shows different kinds of probing instruments used by dentists (b) Bitewing X-ray with an interproximal lesion between teeth 3 and 4 - gold standard for early interproximal caries [3] (c) Patient’s mouth after using a biofilm disclosing agent to see dental biofilm coverage (d) Biofilm micro-environment: pH level is lower moving from surface to enamel [4]. Extracellular Polymeric Substance (EPS) composition and characteristic is shown in the inset figure. (e) Caries formation (f) O-pH in operation at a dental clinic with an inset figure showing a closer look of the device inside the mouth. The tip of the probe used to transmit and collect light is hovering over the occlusal surface of the subject. Detailed description of the device is provided in Fig. 3 and methods and materials section.

CHRONIC caries in teeth, commonly known as tooth decay, is the most prevalent health condition affecting 2.3–3.5 billion people globally [1], [2]. Untreated caries can cause excruciating pain and lead to permanent tooth loss along with adding substantially to a family’s medical expenditure [2]. Presently, visualization and tactile inspection is a standard procedure to evaluate dental surfaces. These techniques are the only gold standard for detecting early caries at occlusal (biting) and smooth surfaces (Fig. 1(a)), while bitewing X-rays (Fig. 1(b)) are the diagnostic tools used for caries at interproximal (in between teeth) regions. Lesion activity is determined by surface roughness and appearance whereas lesion depth is confirmed using X-rays.

These dental tools and procedures provide patients with lagging, non-quantitative feedback assisting inadequately in prevention of new caries or in evaluating site-specific risk of caries development. In spite of oral care playing a significant part of a healthy daily routine, from brushing twice a day, frequent flossing, avoiding foods with excessive sugar, and minimizing snacks in-between meals, in addition to bi-annual dental visits, patients are still unable to evaluate effectiveness of their daily oral-care. Dentists, on the other hand, can’t objectively confirm if the patients, especially adolescents, are performing effectively their daily care routine unless a suspicious spot is clinically evident. There is a need to interject this present cycle of waiting-and-watching for a lesion to appear, in order to evaluate oral well-being using tools that can provide leading indicators for oral health. A leading indicator, a terminology commonly used in occupational health systems [5], provides pro-active, predictive risk assessment unlike lagging tools that assess information after an event has already occurred, particularly in our case, after a carious lesion has formed. Similar to a visit to a general physician where measurements like heart rate, blood pressure, and blood work provide a baseline quantitative information, dentistry could benefit with quantitative measurements of the risk factors that are directly correlated with caries formation and can be safely monitored over time to understand the status of oral health. The current adjunct diagnostic tools are focused on measuring the presence of the disease, rather than assessing the risk of developing active caries.

One of the techniques to obtain quantitative measurement of caries risk is by developing tools to monitor oral enamel biofilm - the sticky, yellowish coating found on teeth surfaces which plays a crucial role in early caries. Presently, dental biofilm (also referred to as plaque) is evaluated using visual quantitative measurement techniques like Quigley Hein plaque index [6] that measures and ranks dental biofilm coverage with help of probing tools but is unable to objectively evaluate cariogenesis of biofilm. Similarly, disclosing dyes (as shown in Fig. 1(c)) assist in visual inspection of dental biofilm, though staining of teeth makes the use uncommon. There are fluorescent based devices like SOPROcare and Q-Ray that capture fluorescence by exciting porphyrin found in oral biofilm [7], [8] with blue light. These devices increase dental biofilm visibility and also indicate dental biofilm maturity which is proportional to the intensity of porphyrin’s red fluorescence. Though these fluorescent devices provide leading indicators, they focus on very specific porphyrin producing bacterial groups (*Streptococcus mutans*, etc.) [9], [10], ignoring the impact of vast number of (over 700) microbes found across different oral cavities [11], [12] and are confounded by food stains, lowering specificity as a stand-alone leading indicator of caries. Several low-cost, at-home, dental biofilm monitoring devices have been proposed, for example, Angelino *et al.* [13] designed Plaquefinder, a low-cost, open-source, ~405 nm LED (Light Emitting Diode) based device, and the associated computer vision algorithm that captured red fluorescence signatures associated with dental biofilm and demonstrated comparable performance to commercially available devices. Similarly, with LumiO, Yoshitani *et al.* [14] added red fluorescence technique to an electric toothbrush custom fitted with a camera to assist in brushing by increasing visibility of dental biofilm. They found qualitative evidence that study participants were able to improve awareness of dental biofilm and build confidence on their toothbrushing. These devices can enable home based dental biofilm index monitoring and aid in practicing oral hygiene but are unable to track acidification of dental biofilm making it less effective in preventing caries formation.

Our mouth with its optimum temperature (35–37°C), neutral pH, and frequent access to nutrients is a breeding ground for several hundred species of micro-organisms, found around tooth surfaces and gum lines [15]. On consumption of carbohydrates, bacteria in the dental biofilm produce acid which is slowly neutralized by the action of saliva. This compensating mechanism can be disturbed with frequent consumption of sugar rich food, lack of proper dental hygiene, disruption in flow of saliva, and other life style habits, increasing the acid production, its frequency, and duration of acid exposure to enamel. This leads to a change in micro-environment favoring growth of harmful bacteria that can survive in low-pH and anaerobic conditions as shown in Fig. 1(d). If left unmonitored without intervention, extended exposure to acid can degrade the tooth enamel of minerals to become a demineralized lesion and ultimately cause carious cavitation as depicted in Fig. 1(e). Thus, routine monitoring of the acid producing function of the biofilm which plays an early critical role in the degradation of enamel can help us understand pH changes as a leading site-specific risk indicator to caries formation.

**Fig. 2.**
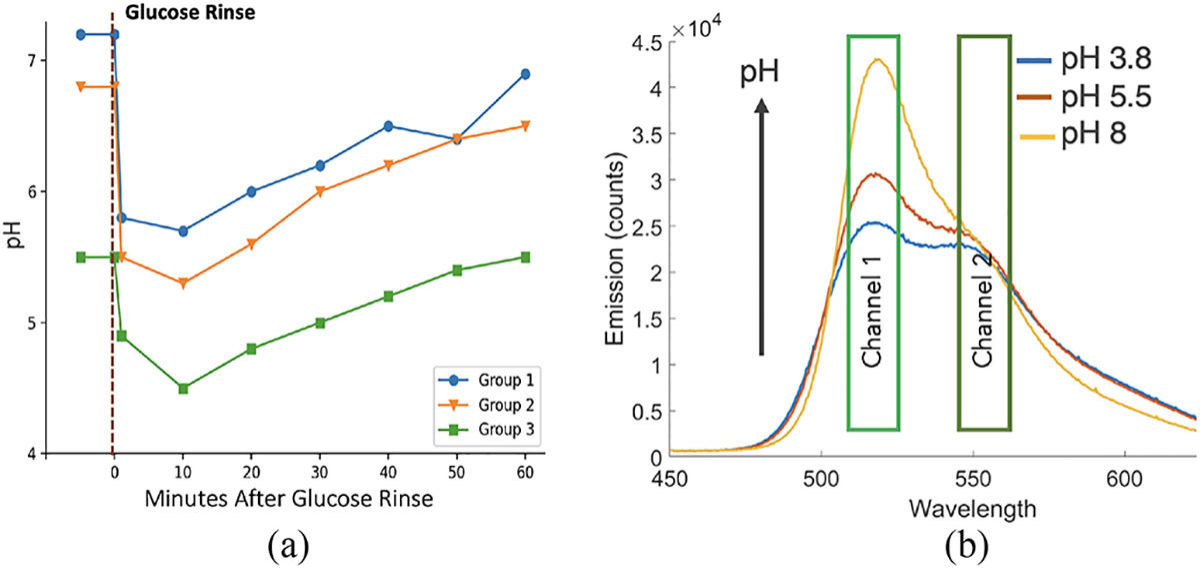
(a) The Stephan curve, pH response of oral film immediately after a sugar rinse and monitored upto 1 hr in three subject groups with different caries risk (Group 1: caries free, 2: slight caries activity, 3: extreme caries activity.) Several studies have shown that drop in pH after sucrose rinse is dependent on caries activity in the region [27]. The graph includes three of the 5 categories of subjects represented in 1944’s Stephan Curve. (b) Fluorescence spectrum of aqueous solution of sodium fluorescein in different pH solutions obtained using 420 nm LED excitation and captured with a spectrometer. O-pH uses peak at 520 and 550 nm to measure pH.

Measurement of dental biofilm pH, especially pH before and up to two hours after a sugar rinse was proposed in 1940s [16] as shown in Fig. 2(a). Since then, several studies have examined this pH curve, commonly named as the Stephan curve, and found different sections of the curve: resting pH [17], [18], minimum pH after the sugar rinse [19], [20], time taken to return to resting pH [21], related to caries activity. Most prior pH studies [22], [23] have used pH micro-electrodes to measure dental biofilm pH. Latest micro-electrodes are only 0.1 mm in diameter making them suitable for many interproximal measurements. But their fine structure and need for a glass reference electrode makes them prone to fragility, breakage, and inconvenience [24]. Recently, pH strips were used to measure pH at interproximal sites and found to have high correlation with electrode based pH measurement [25], [26]. These pH strips are a low-cost alternative to micro-electrode system, but are difficult to insert at interproximal spots without wedging and unable to measure in deeply pitted occlusal surfaces. Additionally, both the pH strip and the pH micro-electrode are contact based method and can disrupt the biofilm during measurement. They also measure pH at the dental biofilm-saliva interface [24] which doesn’t represent the pH of dental biofilm matrix. Therefore, there is a need for development of devices and protocols that can easily and comprehensively measure dental biofilm acidity in the clinic.

In this work, we present O-pH, an optical pH-sensor [Fig. 1(f)], that uses ~420 nm light to excite fluorescein dye and collects fluorescent light using fiber coupled, filtered photodiodes. It measures pH in the range of 4–7.5, typical pH range of the dental biofilm, with 0.97 coefficient of correlation to a standard lab pH-meter. The device was tested on 30 high caries risk pediatric subjects to understand clinical relevance of dental biofilm pH and to develop a clinically relevant protocol that fits within standard workflow. The device was tested in a standard clinical setting to measure oral biofilm pH before and after a sugar rinse. Testing was performed on two groups, one that had a professional dental cleaning within last three months and a second group that didn’t have a professional cleaning for more than three months. The current point-based device can be extended to image based sensing that reduces *in vivo* measurement variability by co-registration of pH mappings and a case study with the prototype is also presented.

## II. MATERIALS AND METHODS

### A. Sodium Fluorescein Properties

Sodium Fluorescein (Fl), is a dye commonly used as diagnostic tool in ophthalmology and approved by FDA for human use. In the aqueous solution it has a peak absorption band at ~490 nm and fluoresces with a wide spectra from 500 to 650 nm with a distinct peak at 520 nm. This emission intensity is directly proportional to the extracellular biofilm pH. Additionally, Fl has been shown to rapidly penetrate dental biofilm extracellular matrix making it an ideal candidate for pH measurement of dental biofilm [28]–[30].

Sjoback *et al.* [31] have shown that in aqueous solution, Fl exhibits an equilibrium mixture of four different species: cation, neutral, anion, and dianion. Out of the four, only the dianion and anion species are fluorescent, having different absorption and emission peak, and pH dependent concentration in the solution. For example, at pH 4 and lower, a Fl solution consists of predominantly anions, and at a pH 9, the solution mainly has dianions resulting in different spectral properties in the 450–650 nm range [32]. Solutions between pH 4–7.5 contain both dianion and anion species resulting in a fluorescent spectral profile that is a mixture of individual emission profiles [Fig. 2(b)] distinctly observed by selecting an excitation wavelength that can excite both species (~420 nm). As previously demonstrated, Fl emission spectra captured using spectrometer can be unmixed with least mean square to predict pH [32]. Our prototype, O-pH, uses distinct fluorescence properties of Fl dianions and anions species, but instead of using the entire spectra, it utilises only the two peaks at 520 and 550 nm to calculate pH in the range of 4–7.5 [Fig. 2(b)].

### B. O-pH: Device Architecture

The device architecture consists of three components: (a) excitation unit (b) detection unit (c) mouth probe.

**Fig. 3.**
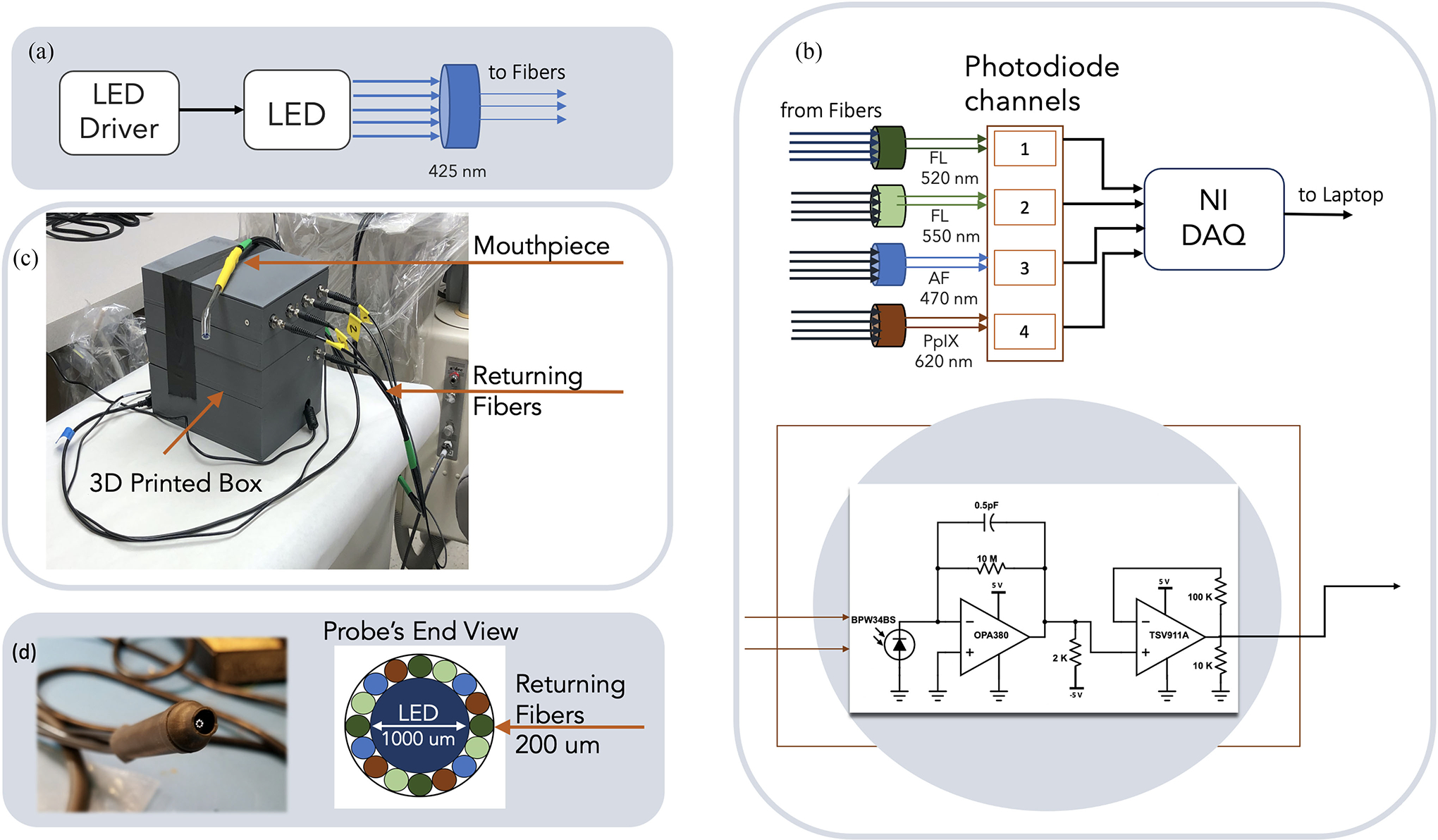
Device Architecture: (a) Excitation Unit (b) Photodetector Unit [FL: Fluorescein, AF: Auto Fluorescence, PpIX: Porphyrin] with schematic for photodiode channel (c) 3-D printed box with optical fibers attached (d) Fiber optics probe and its end view.

The excitation unit is used to excite the Fl solution and comprises a LED driver (Thorlabs, LEDD1B) pulsing a blue LED (ThorLabs, M420F1) at 500 Hz with 5 W. The pulsing LED light is filtered using a fluorescence, band pass filter (Semrock, FF01–425/26–25) centered at 425 nm to limit the bandwidth of the excitation wavelength (Fig. 3(a)) and block out-of-band emissions [33].

The emitted fluorescence on absorption of LED light is measured using the detector unit which consists of four independent, optically filtered, photodiode channels Fig. 3(b). Different channels of the detector unit are used to capture Fl fluorescence and low signal emissions. Channels 1 and 2 of the photodiode board is used to detect Fl anion and dianion fluorescence intensity. Channel 1 uses a band-pass filter (BP) centered at ~520 nm (Semrock, FF01–524/24–25) to measure emitted photons from dianions and Channel 2 uses a BP filter centered at ~550 nm (Semrock, FF01–549/12–25) to measure emission from anions. Channels 3 and 4 are used to detect low level fluorescence in the mouth that can be excited by the 420 nm LED light, namely autofluorescence (AF) and porphyrin’s (PpiX) fluorescence. These channels use a filter centered at 475 nm (Semrock, FF02–475/20–25) and another centered at 632 nm (Semrock, FF02–632/22–25) for AF and PpiX respectively. Each photodiode circuit, shown in Fig. 3(b), consists of a Silicon photodiode (BPW34BS) where the incoming photon is collected, generating current which is then converted to voltage using a transimpedance amplifier (TI, OPA380) with a gain of 10 M V/A. The output voltage of the transimpedance amplifier is amplified using a non-inverting amplifier (TSV911 A) with a gain of 11 V/V. The final output voltage is sampled using National Instrument’s data acquisition unit (NI, DAQ600) at 10 KHz frequency.

The above two units are housed inside a 3D printed box, shown in the Fig. 3(c) with jacketed optical fibers coming out of the box. The fiber optics bundle, consists of central 1000 *μ*m fiber (ESKA, Mitsubishi) that carries the excitation light from the LED, and surrounded by sixteen returning 200 *μ*m fibers carrying the emitted fluorescent light to photodiodes. Each photodiode channel inside the box is coupled to four optical fibers to receive emitted photons. The length of all fibers is one meter to provide flexibility for the operator to probe far back in the mouth with the device. These fibers terminate in a hand-held dental probe; such that the tip of the probe has the excitation fiber in the center surrounded by returning sixteen fibers in a circular ring. Fig. 3(d) shows the image of the probe’s tip and it’s end view. A rubber barrier is used at the tip of the probe to avoid physically touching the fibers tip to subject’s teeth and is changed for every subject.

### C. O-pH: Algorithm

The sampled voltages from the DAQ is transformed to frequency domain using Fast Fourier Transform. The amplitude of signal corresponding to 500 Hz is recorded for each photodiode channel. This is the frequency of the pulsing blue LED and selecting the voltage amplitude corresponding to this frequency helps in discriminating against background light. Extracted fluorescence reading from channel 1 and channel 2 is then used to calculate pH. Channel 3 recording is utilized to measure the AF noise which acts as a threshold to accept or reject estimated pH. This threshold is estimated during the calibration process. Channel 4 data is used to measure PpiX fluorescence as another indicator of dental health.

### D. O-pH: Device Calibration

O-pH requires a one time calibration for pH measurement. We describe the calibration process and device accuracy in subsequent sections.

#### 1) Chemical Preparation:

1 Molar stock solution of sodium fluorescein (Sigma Aldrich and ScienceLab) was prepared in deionized water. The fluorescein solution was diluted in phosphate citrate buffer (0.2 M dibasic sodium phosphate, 0.1 M citric acid, pH indicated for each experiment), 0.1 M sodium bicarbonate buffer, or chemically defined medium (CDM) buffer to form solutions in the range of 4 to 7.5 pH [32]. These solutions of 200 *μM* concentration were used for calibration of pH device with a conventional pH meter (ThermoFisher Scientific).

#### 2) Fluorescence Measurement:

**Fig. 4.**
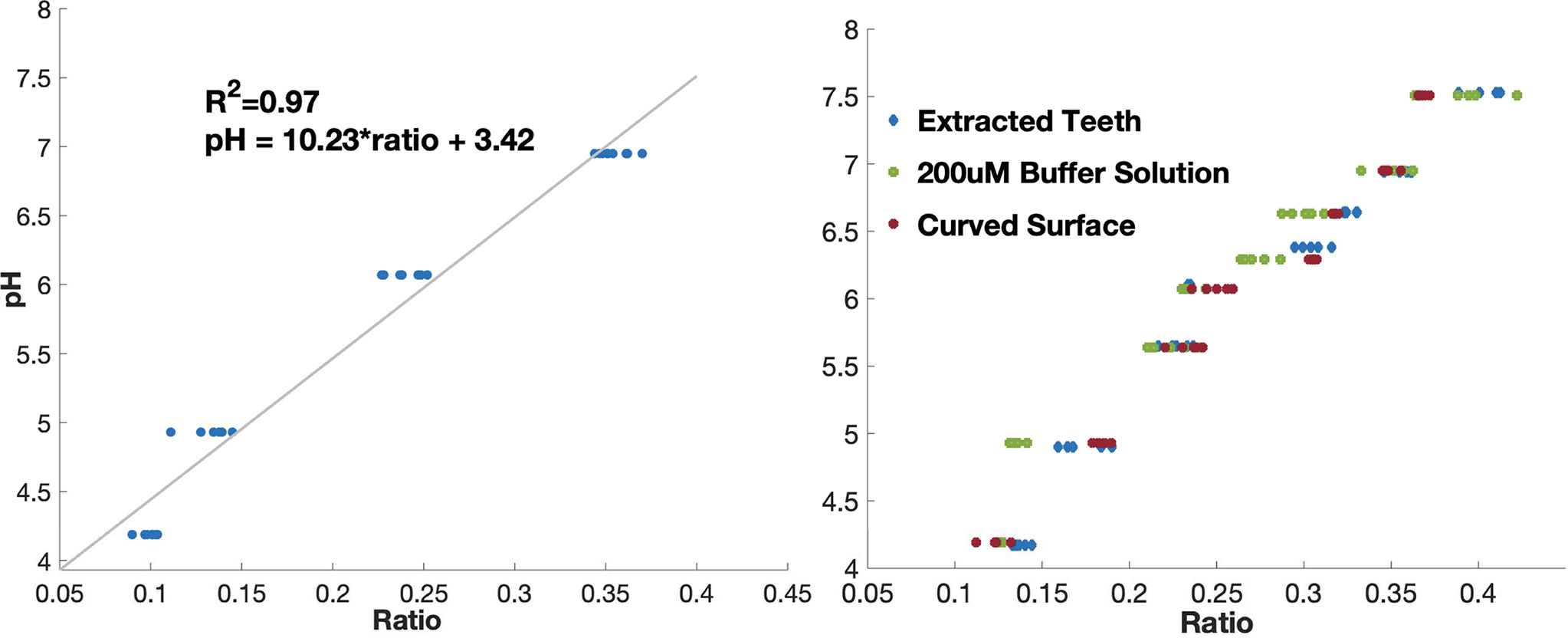
(a) Calibration curve using buffer solution in a 1 mm cuvette. Ratio is given by 1. (b) Verification of calibration curve using 200 uM buffered fluorescein in 1 mm cuvette, on extracted human teeth, and on artificial curved teeth surfaces (occlusal, interproximal, and buccal surfaces of artificial teeth). A drop of fluorescein is added on different teeth surfaces and pH is measured using O-pH.

Using a 1 mm glass cuvette, we measured fluorescence of 10*μL* of four different 200 *μM* Fl buffers ranging from pH 4 to pH 7.5. Each measurement was repeated ten times to obtain the calibration curve as shown in Fig. 4(a). A linear relationship was obtained between pH and ratio defined in 1 with a correlation coefficient of 0.97.


(1) $$ratio=\frac{Ch1-Ch2}{Ch1+Ch2}$$

(2) $$pH=10.34*\frac{Ch1-Ch2}{Ch1+Ch2}+3.42$$

We verified the calibration curve by measuring different Fl buffers in the same pH range using the 1 mm cuvette used in calibration. Since the calibration curve was obtained using a flat surface but *in vivo* testing would be performed on irregular surfaces, so the device was verified on artificial teeth surfaces (Perio 525 Typodont, frasaco GmbH). We dispensed Fl on occlusal, interproximal, and buccal surfaces to measure pH values. Next, we tested Fl on extracted human teeth to see the effect of low signal levels of AF. We found the pH measurement was robust to AF if the AF signal is below a threshold. This threshold was noted and used in clinical testing to discard measurements. All the predicted pH values are plotted in Fig. 4(b), obtaining an overall correlation coefficient of 0.92. The device had an overall error of 0.22 pH with 0.16 standard deviation. O-pH device accuracy in various pH ranges are listed in TABLE I. We found that fluorescence readings of channel 1 and channel 2 made inaccurate predictions if the fluorescence was too low, but this signal could be amplified by increasing the excitation power. Distance of the probe from measuring surface doesn’t affect the accuracy if the fluorescence signal strength is above this threshold. With our maximum current and voltage setting, we found that at a separation distance up to 3 mm, the device probe provided accurate results. To note, we bounded our measurements between 4 and 7.5 pH, discarding any values outside this range as inaccurate.

### E. O-pH: Clinical Study

The clinical study, the first optical based pH measurement of dental biofilm, was designed with pediatric patients to monitor dental biofilm pH before and after a sugar rinse for both healthy and unhealthy teeth surfaces.

#### 1) Recruitment:

Pediatric patients catagorized as high caries risk after clinical exam at University of Washington’s Center of Pediatric Dentistry (CPD) were recruited along with a control group comprised of low caries risk patients. The inclusion criteria for the high caries risk group include at least one active lesion (cavitated or non-cavitated) either at interproximal region between maxillary posterior teeth, or at occlusal surface of mandibular posterior teeth. The inclusion criteria for the low risk control group included absence of active caries lesion or any existing restorations.

We excluded subjects undergoing active orthodontic treatment at study selected sites, having asthma, eczema or any known allergy to yellow dyes. The high risk group is further divided into “Post-Cleaning group” and “Pre-Cleaning group” based on their recent history of professional dental cleaning. A total of 30 subjects were recruited, the “Post-Cleaning group” (n = 18) has subjects with professional dental cleaning within last three months, the “Pre-Cleaning group” (n = 7) has subjects without professional dental cleaning for over 3 months, and lastly, a control group with subjects in low-caries risk category and a professional dental cleaning within three weeks (n = 5), see Table II. Subjects were given a remuneration gift card for participating in the study and the study was approved under our institution’s IRB (IRB ID: STUDY00007002).

#### 2) Protocol:

The study protocol used ICDAS II ranking scheme to rank maxillary interproximal and mandibular occlusal surfaces [34], performed by a dentist at CPD using bitewing radiographs and clinical exam charting at a routine patient visit. Ranking was performed three weeks before the O-pH appointment for the Post-Clean group and within a week after the O-pH appointment for Pre-Clean group. Additionally, all teeth surfaces with no caries activity were ranked as 0 and with any carious lesion as 1, giving us a binary distinction between teeth surfaces. For every subject, we had a high number of 0 ranked tooth surfaces and only a few ranked 1. There was a minimum interval of three weeks between cleaning and pH measurements using O-pH for the Post-Clean group to allow the dental biofilm to mature.

At O-pH testing in the University’s dental clinic, third and second year dental students (n = 5) performed the pH measurements under the supervision of a dental faculty. The dental students were aware of the inclusion/exclusion criteria but blinded to group designation and surface rankings. Before the measurement, subjects were asked to rinse their oral cavity with water. Subjects were asked to produce 10 mL of saliva in a measuring cup and it’s pH was measured using a conventional pH meter, followed by a baseline measurement of test surfaces (maxillary interproximal and mandibular teeth occlusal surfaces) to detect teeth AF. Next, we measured, the “rest pH” after applying Fl on the same set of teeth surfaces using a blunt hyperdermic needle one tooth at a time. Subjects then retained 10 ml of 0.3 M sucrose solution in their oral cavity for fifteen seconds. They were instructed to either swallow or spit out the sucrose solution. One minute after the sugar rinse, we measured the “drop pH” by re-applying Fl. Difference between rest pH and drop pH was calculated and called “diff pH”. Application of fluorescein and pH measurement at each spot took a few seconds. At maximum, it took an additional two minutes between the measurement of first and last tooth. Each set of pH measurements (rest, drop pH) were taken with mouth open, but patients were allowed to close their mouth or speak in between measurements if it was too uncomfortable. Each measurement with O-pH at a tooth surface was repeated thrice and average of the three was used for analysis. Subjects were not provided with any prior instructions on skipping meals or to avoid brushing. Since, saliva pH is generally neutral across subjects, we used it as a stable baseline to normalize pH values across subjects. For analysis, we normalized rest and drop pH w.r.t to saliva pH and compared across different surfaces. This is an additional metric that we looked at as it takes in account impact of saliva on caries formation.

#### 3) Statistical Analysis:

To measure variability in device measurement, we collected three readings per spot for rest and drop pH. Each triplet’s mean and standard deviation were used to calculate the pool standard deviation of the device. This gives the average spread of all data points about their group (triplet) mean. For clinical data analysis, groups with normal distributions but unequal amount of data (pH measurements of Post vs Pre-Cleaning group) were compared using Welch’s t-test [35] and permutation test [36] at 0.05 significance level. In case of groups without a normal distribution (pH of Pre/Post Cleaning group having surfaces with rank 1), only permutation test was used for significance analysis. Shapiro-Wilk’s normality test was used to test normal distribution of data distribution of data [37]. Different Groups and the statistical tests used are elaborated in the Results section. All analyses were performed using SciPy library in Python.

### F. Non-Contact pH Imaging

#### 1) Device Design and Calibration:

With the present spot based system it is difficult to perform trend analysis over short times for the Stephan Curve within a single visit, let alone months-long gaps in time across multiple visits. These challenges can be overcome by using an imaging system, image co-registration, and an improved clinical protocol. To demonstrate this concept, we modified the multi-modal Scanning Fiber Endoscope (mm-SFE) to use the two wavelength technique employed by O-pH for optical pH image-based mapping. The mmSFE scans the distal end of a single 80 *μ*m diameter optical fiber in a spiral pattern at 10–12 KHz using a custom tubular piezoelectric actuator and a custom lens assembly [38]. The vibrating singlemode fiber emits 424 nm light (Nichia laser diode with Thor Labs Fiberport and clean up Semrock Brightline bandpass filter at 420+/−5 nm) that is nearly collimated for a forward view from the mmSFE tip. By collecting backscattered reflectance (B-channel) and emitted fluorescence channels (G channel centered at 520 nm and R channel centered at 549 nm) in a ring of multimode plastic optical fibers, three spectral bands of RGB are created after filtering and photomultiplier detection [39]. Similar to O-pH, we verified the imaging based device *in vitro* and built a calibration curve using the ratio, (G−R)/(G+R) w.r.t to pH. The relationship for each pH value was obtained by averaging 10 video frames acquired over 10 seconds [39].

#### 2) Protocol:

A low-caries risk subject without a professional cleaning in last seven months was examined using the O-pH-scope after skipping brushing for 5 days. We used the modified protocol from the clinical study to enable faster measurement. Instead of applying Fl with syringe one tooth at a time, subject rinsed mouth with Fl before resting and drop pH mea-surement (Fig. 7). This study was approved under our institution’s IRB (IRB ID: STUDY00002579)

## III. RESULTS

### 1) Device Verification:

In the clinic, we relied on the device accuracy from *in vitro* testing and verified whether the device can take repeatable measurements. In total we measured rest pH at 85 surfaces and drop pH values at 95 surfaces, giving us a total of 180 readings. Since, each reading was measured thrice, we had a total of 540 readings. For a few measurements (<1%), we had lesser than three readings, as data points had to be discarded because of low quality or out of range pH prediction. To verify repeatability, we calculated mean and standard deviation of each rest/drop measurement triplet and then calculated pooled standard deviation. We obtained 0.23 pH of pooled standard deviation with our data, i.e. the actual readings were within 0.23 pH from the measured mean value of a triplet. Lack of clinically approved oral pH measurement devices hindered us from verifying the accuracy of the device *in vivo*.

### 2) Clinical Findings:

Assuming Pre-Clean group has higher dental biofilm level, we analyzed Pre-Clean and Post-Clean group to understand differences in pH measurements. The control group comprising caries free subjects was tested within three weeks of professional dental cleaning and lacked significant biofilm growth resulting in reduced Fl absorbance and low fluorescence emission for pH detection. The result helped us modify the clinical protocol to maintain at least a three week interval between professional cleaning and testing.

**Fig. 5.**
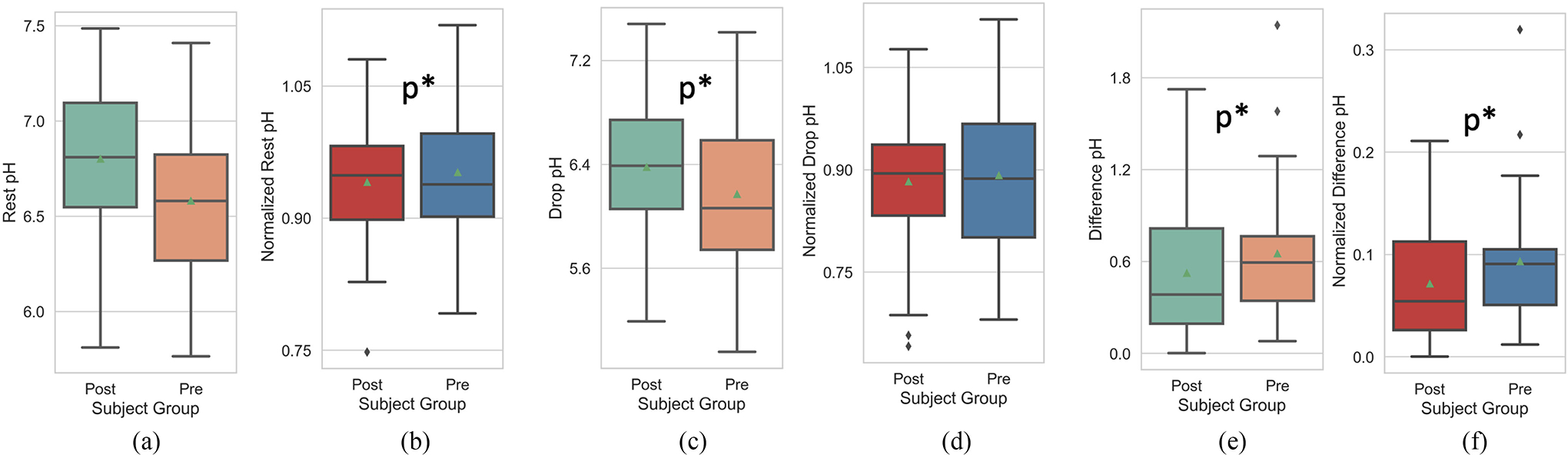
Box plots of Post and Pre Cleaning group for (a) Rest pH (b) Saliva normalized Rest pH (c) Drop pH (d) Saliva normalized Drop pH (e) Difference pH (f) Saliva normalized Difference pH with p* indicating significance with p<0.05.

**Fig. 6.**
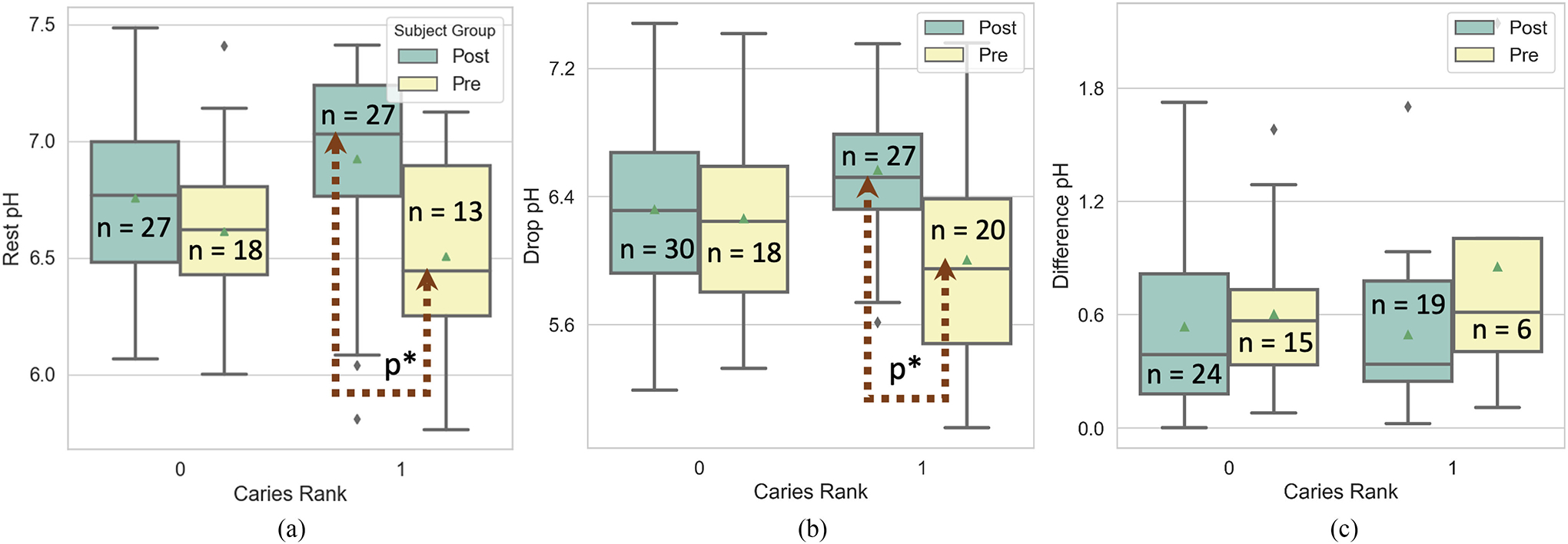
Box plot of pH measurements for different ranks per group using (a) Rest pH (b) Drop pH (c) Difference pH, with p* indicating significance with p<0.05 and n = number of teeth surfaces measured.

We hypothesise that lower rest and drop pH, and higher diff pH, are associated with higher level of “unhealthy” dental biofilm contributing to elevated caries risk in a certain subject. To test this hypothesis, we compared the resting, drop and difference of pH obtained between the two recruited groups. We found Pre-Cleaning group had a lower resting and drop pH than the Post-Cleaning group. Similarly, the difference in pH was higher in Pre-cleaning group than the Post-Clean indicating higher bacterial acidification. Fig. 5(a),(c),(e) shows the distribution of rest pH, drop pH, and diff pH obtained in the two groups. Since, we had unequal number of data in each group, we used Welch’s t-test and permutation test to measure if the pH differences between the two groups were significant. We found that drop pH was significantly lower (alpha < 0.05) in Pre-Cleaning compared to Post-Cleaning with p = 0.0008 using both tests, diff pH was significant only using permutation test (p = 0.014). The rest pH was lower for Pre-Clean group but we didn’t find significant difference. We also compared pH between groups with the same ranking, i.e., surfaces with rank 0 in Pre-/Post-Cleaning were compared and did not find any significant difference. For subjects with rank 1, rest pH and drop pH had a significant difference with p = 0.004 and 0.003 respectively using permutation test (data did not have a normal distribution). Fig. 6(a),(b),(c), shows distribution for both ranks along with number of teeth surfaces measured.

Next, comparing saliva pH between the two groups, it was observed that Pre-Clean had a lower pH than the Post-Clean group though average difference was not significant. On normalizing pH measurements with subject’s saliva pH (measured before the sugar rinse), significant difference was obtained for rest pH (Welch’s t-test, p = 0.003) and diff pH (Permutation t-test, p = 0.014), see Fig. 5(b),(d),(f). Since, the data is normalized using saliva pH, it is difficult to predict the direction of the difference unlike pH measurements in Fig. 5(a),(c),(e) where a low “rest” or “drop” pH means higher acidity. For rank based normalized pH analysis for each group, we did not find any significant difference.

We also examined all the subjects irrespective of the cleaning group to see difference between caries and non-caries surfaces. We found average rest, drop, and diff pH for non-caries surfaces are : 6.73, 6.3, and 0.55 whereas for caries surfaces are : 6.81, 6.36, 0.56 respectively.

### 3) Non-Contact pH Imaging:

**Fig. 7.**
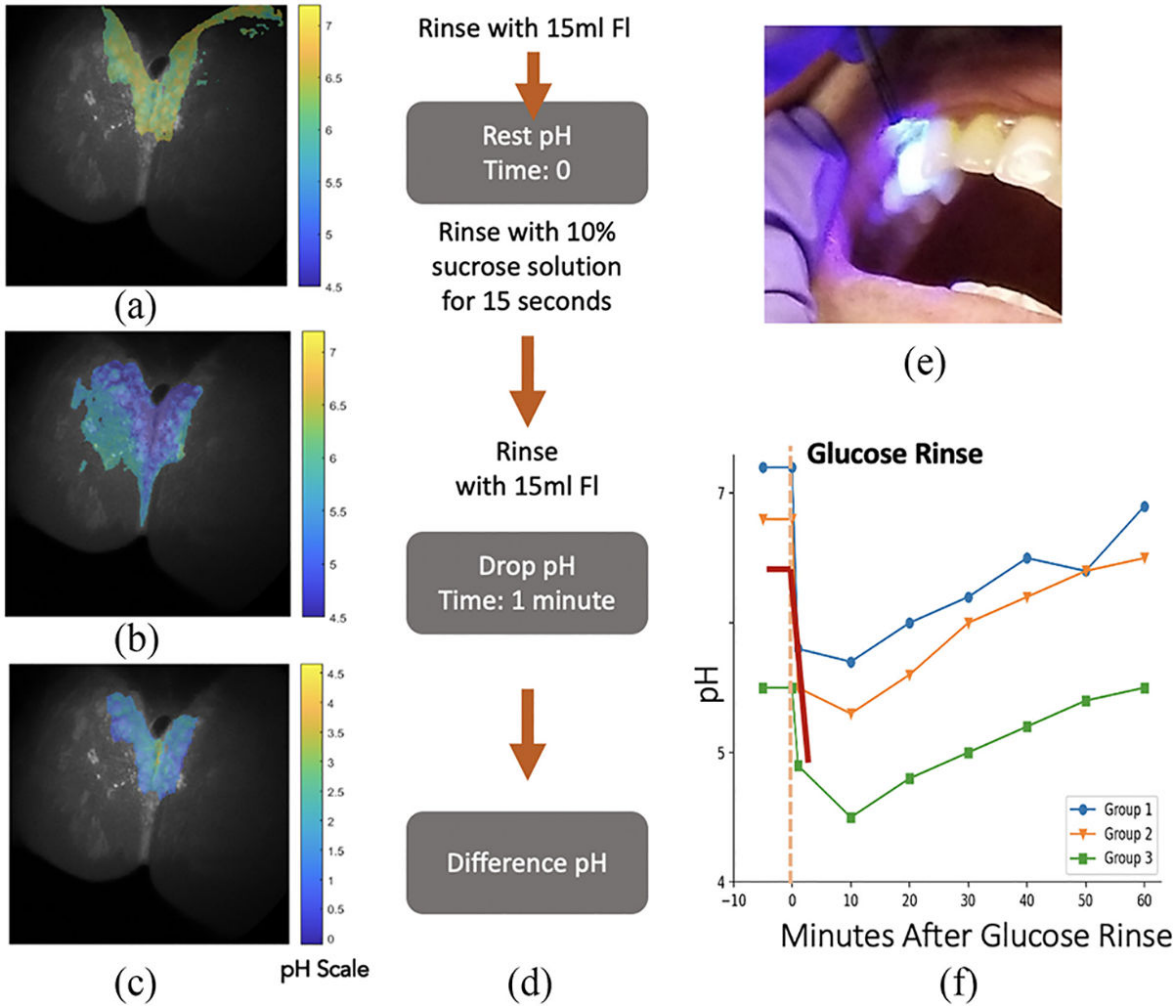
Case study with mm-SFE based pH sensing. The subject had not received professional cleaning for over seven months and had skipped brushing for 5 days prior to the examination. (a) Interproximal dental biofilm image with pH heatmap (b) pH heatmap after a sugar rinse (c) Difference between resting and drop pH (d) Protocol used for testing with mm-SFE. Fluorescein is rinsed instead of applied on each tooth surface using a blunt hyperdermic needle unlike the previous clinical study (e) mm-SFE pH probe (f) Stephan curve with red line indicating the average pH obtained using images at each stage. Group 1 to 3 are same as Fig. 2(a).

The reflectance image of teeth overlaid with pH information enables tracking of regions before and after the sugar region. As shown in the images, rest pH around 6.4–7 was obtained, with 5–5.5 drop pH, and diff pH around 1.5 pH, similar to group 2 of Stephan’s study (Fig. 7(f)).

## IV. DISCUSSION

In terms of measuring capability, the device performed best in the Pre-Cleaning group in comparison to other groups as we measured 40 surfaces amongst 8 subjects whereas only 45 surfaces across 18 subjects in the Post-Cleaning group. Higher Fl fluorescence signal in Pre-Cleaning group along with lower AF signal assisted in obtaining repeatable measurements. We measured at least 4–5 surfaces per subject but many readings in Post-Cleaning group were discarded because of high AF, indicating fluorescence by enamel or underlying tissues. Presence of higher AF in Post-Cleaning group vs Pre-Cleaning group could be indicative of thinner dental biofilm coverage resulting in capture of higher fluorescence from enamel. Across both groups, we noticed that surfaces to which fluorescein application was convenient, for example, upper-distal-interproximal, and lower-occlusal surfaces, had a higher signal to noise ratio. Drop pH values were more repeatable than rest pH value and perhaps the combination of sugar and fluorescein made the dye adhere to the biofilm more. Biofilm index (Quigley Hein plaque index) of teeth surfaces weren’t measured but we observed that areas with low growth of biofilm had higher auto fluorescence signal. The device algorithm was found to be robust to clinical light settings. The linear fit for calibration does cause lower accuracy in lower pH range (pH 4–4.5, TABLE I) but avoids overfitting of curve. To make device robust to noisy fluorescence, we decided to use AF as a threshold to discard pH measurements, but future versions can be built to adjust the calibration curve based on captured AF signal.

Mean rest/drop pH values of healthy/unhealthy surfaces were comparable on combining both the Post and Pre-cleaning group data. In the Pre-Cleaning group, which consists of a typical patient at a dentist’s clinic for a routine recare visit, resting pH and drop pH (pH after the sugar rinse) for unhealthy surfaces (rank 1) are lower than the healthy surfaces (rank 0), though larger studies are needed to show significance. Population based standard levels of rest and drop pH could be established using clinical studies to help dentists/patients evaluate oral health quantitatively. The pH trend was opposite in Post-Cleaning group. Though this seems contrary to popular cariology concepts, prior studies have shown a wide range of variation in pH profile for unhealthy and sound enamel. P. Lingström’ et.al [20] measured similar rest pH and drop pH at sound and white spot regions. In another study of sound and carious (past the early caries stage) root surfaces in the same subjects yielded indistinguishable biofilm pH profiles [40]. A number of reasons could have caused the confounding results in our case, for example, it’s possible that the Post-Cleaning group perhaps isn’t representative of ‘true enamel environment’ as it consists of young dental biofilm, resulting in a pH profile different from Pre-Cleaning group. Additionally, subjects in Post-Cleaning group were informed three weeks prior to the O-pH appointment about presence of unhealthy/carious surfaces. This could have prompted some of the subjects to improve their oral hygiene preventing build-up of harmful biofilm. The amount of dental biofilm in the Pre-Cleaning group is generally higher than the Post-cleaning group but it is not the amount but the composition of biofilm that plays critical role in caries formation. Unfortunately, the study didn’t include microbial analysis of biofilm and we need further studies to confirm whether both young and mature biofilm at unhealthy surface has different bacterial profile or not. If the profile is indeed different, it will further strengthen the need of a pH monitoring device in clinic as it can measure ‘present’ biofilm activity and aid as a tool to assess oral hygiene.

The significant difference of drop and diff pH in Pre- vs Post- Cleaning group (Fig. 5(c), (e)) indicates that O-pH could be used in the dental clinic as a hygiene tool to measure the growth of acid producing dental biofilm. It can also be useful as an educative tool to help patients, younger patients in particular, understand the immediate harmful impact of sugar rich diets on mouth’s micro-environments and assert importance of professional dental cleaning. In comparison to Stephan’s 1944 study [27], we obtained a smaller average diff pH (0.84 and 0.48 for Pre- and Post- respectively, Fig. 6(c)), lower than 1 pH unit for caries surfaces. The diff pH was similar to difference reported in Lingström’s 2000 study [20] between sound and white spot lesions. One of the reasons could be the averaging technique, Stephan’s study had categories with different caries activity and reading was averaged across all surfaces (sound and unhealthy surfaces) per category but the Lingström study looked at difference between sound and white spot surfaces and averaged only for similar surfaces, similar to analysis represented in Fig. 6. We haven’t used any subject based averaging as that reduces teeth/surface specificity. Though our study analyzed both carious and caries-free surfaces from same subjects, it lacks evaluation using contralateral surfaces in the oral cavity. Additionally, to have sufficient enrollment we did not advise subjects to skip oral routines (brushing, flossing, etc.) or increase intake of sugar. Recruiting subjects who have abstained from brushing for couple of days and sub-dividing them into groups of low and high sugar consumption would have helped in better understanding impact of sugar as well as oral-hygiene on pH.

O-pH requires moderate biofilm build up to measure pH with high signal to noise ratio as indicated from the lack of sensitive measurement in the control group. This is a device limitation that it needs medium/high biofilm deposit to measure pH and can be improved using higher excitation power and Fl concentration. Interestingly, prior studies [25], [26], [41] have had subjects skip brushing for 1–3 days to obtain Stephan curve with biofilm mass above 0.5–0.75 mg per site to have reproducible results [20]. This indicates that higher level of biofilm build up is needed to differentiate between healthy/unhealthy surfaces using acidity monitoring.

Further, as saliva pH also plays a role in caries formation [42], another metric, normalized pH measurements (biofilm pH/saliva pH), was used to understand if the trend is different for (healthy/unhealthy) surfaces and found results similar to non-normalized data. Though, saliva pH is an important factor to consider, normalized pH takes away the intuitiveness of biofilm pH as an acidity indicator.

The accuracy of O-pH was verified with *in vitro* studies using buffered fluorescein solutions and pH meter. *In vitro* study to understand sugar response using lab grown biofilm was not performed. Alternatively, resting pH could be verified *in vitro* by collecting biofilm from the subject’s mouth and measuring pH after dilution with water. But this method was not adopted as it would have caused disruption of dental-biofilm and also reduced the number of spots to measure drop pH in the mouth. Lack of micro-electrodes approved for intra-oral use in United States limited our study from verifying O-pH’s accuracy *in vivo*. Micro-electrodes measure pH at the saliva/biofilm interface and isn’t an ideal ground truth for O-pH that measures pH of extracellular oral biofilm. Microelectrodes, as previously mentioned is a contact based approach and could have caused disturbance in the biofilm impacting readings with O-pH. Therefore our approach for verifying the O-pH performance *in vivo* was based on comparison to prior studies that used pH measurement systems in research settings, as well as demonstrating acceptable repeatability of multiple measurements from the device.

Although O-pH has the potential to be non-contact and thus nondestructive to the dental biofilm, this current spot-based pH sensing has clear drawbacks, especially in reliably testing the same spot before and after a sugar rinse. The lack of replicability in probe placement directly impacts the accuracy of pH drop measurements and has been identified as a source of variability in previous microelectrode measurements [17]. Imaging plays an important role in mapping as dental biofilm pH is highly variable spatially and is a critical enhancement for measuring pH difference. Furthermore, the mmSFE system uses highly sensitive photomultiplier optical detection which may provide sensitive pH sensing with thinner and less mature biofilms. But, the imaging system poses its own image processing challenges because enamel surfaces lack features, making it difficult to align and stitch images. Additionally, air bubbles in mmSFE images hindered accurate pH measurements in the pilot study. However, this challenge may be overcomed in the dental clinic by using compressed air to remove air bubbles. In addition, optical imaging system equipped in some dental offices can create full 3-D images of teeth thus reducing challenges in registering images taken over time. Upcoming hyperspectral cameras can be ultilized instead of mm-SFE to map and measure oral pH [43].

The clinical protocol suggested can be further improved and validated in larger studies. For example, the level of 10% sucrose solution used for the O-pH and mmSFE case study could be raised to 20% sucrose concentration which shown by Lingström’s *et al.* [20] results in higher diff pH. In another example, several studies [17], [20], [44] have shown that at times it may take up to 5 mins to reach the lowest pH after a sugar rinse. So monitoring the drop pH every minute for 5 minutes can perhaps give a better pH differentiation between caries and sound enamel surfaces. We avoided measuring the entire Stephan curve because it would be difficult to implement a testing protocol that lasts 60–90 minutes in routine clinical practice.

## V. CONCLUSION

O-pH measures acidification of the dental biofilm which is a critical step in the caries process, unlike indirect optical methods that rely on the presence of specific bacterial species in the biofilm. The device is capable of measuring pH of biofilm at occlusal pits and fissures and interproximal surfaces with repeatable measurements. Fast diffusion of sodium fluorescein dye into the biofilm enables measurement of pH inside the biofilm’s micro-environment rather than pH on the saliva surface. Additionally, the dye-based methodology allows measurement of extracellular pH without disturbing the biofilm. The initial clinical study with 30 subjects has shown O-pH’s capability to differentiate between low and high biofilm load in subjects using pH measurements. Future studies are needed to confirm its utility as a hygiene monitoring device and to measure pH trends within groups with low plaque load. We noticed, one of the drawbacks of a point-based device was uncertainty of probing the same region before and after a sugar rinse. This limitation was addressed by proposing an imaging-based pH monitoring device developed on the same principle as O-pH and tested on one subject. mm-SFE scope results indicated its ability to track rest and drop pH with images. Further clinical studies are needed to evaluate its usability, sensitivity, and accuracy. O-pH and mm-SFE scope are a step towards development of tools that can break the cycle of lagging dental indicators by providing site-specific trends that monitors direct bio-chemical properties affecting enamel health.

## Figures and Tables

**TABLE I:** O-pH Accuracy

pH Range	Mean Error	Std Deviation

4–4.5	0.57	0.09
4.5–5.5	0.27	0.15
5.5–6.5	0.18	0.09
6.5–7.5	0.13	0.08
Overall(4–7.5)	0.22	0.16

**TABLE II:** Subject Statistics

Subjects	Post-Cleaning	Pre-Cleaning	Control

Total	18	7	5
Age	16.5	15	15
Mean Cleaning Interval	31 days	114 days	14 days
